# COVID-19 and Bone Loss: A Review of Risk Factors, Mechanisms, and Future Directions

**DOI:** 10.1007/s11914-023-00842-2

**Published:** 2024-01-15

**Authors:** Amy Creecy, Olatundun D. Awosanya, Alexander Harris, Xian Qiao, Marie Ozanne, Angela J. Toepp, Melissa A. Kacena, Thomas McCune

**Affiliations:** 1 Department of Orthopaedic Surgery, Indiana University School of Medicine, Indianapolis, IN USA; 2 Critical Care, and Sleep Specialists, SMG Pulmonary, Norfolk, VA USA; 3 Division of Pulmonary and Critical Care Medicine, Eastern Virginia Medical School, Norfolk, VA USA; 4 Department of Internal Medicine, Eastern Virginia Medical School, Norfolk, VA USA; 5 Department of Mathematics and Statistics, Mount Holyoke College, South Hadley, MA USA; 6 Enterprise Analytics, Sentara Health, Virginia Beach, VA USA; 7 Richard L. Roudebush VA Medical Center, Indianapolis, IN USA; 8 Division of Nephrology, Eastern Virginia Medical School, Norfolk, VA USA

**Keywords:** SARS-CoV-2, COVID-19, Muscle, Bone, AI, Artificial Intelligence, ChatGPT

## Abstract

**Purpose of Review:**

SARS-CoV-2 drove the catastrophic global phenomenon of the COVID-19 pandemic resulting in a multitude of systemic health issues, including bone loss. The purpose of this review is to summarize recent findings related to bone loss and potential mechanisms.

**Recent Findings:**

The early clinical evidence indicates an increase in vertebral fractures, hypocalcemia, vitamin D deficiencies, and a loss in BMD among COVID-19 patients. Additionally, lower BMD is associated with more severe SARS-CoV-2 infection. Preclinical models have shown bone loss and increased osteoclastogenesis. The bone loss associated with SARS-CoV-2 infection could be the result of many factors that directly affect the bone such as higher inflammation, activation of the NLRP3 inflammasome, recruitment of Th17 cells, the hypoxic environment, and changes in RANKL/OPG signaling. Additionally, SARS-CoV-2 infection can exert indirect effects on the skeleton, as mechanical unloading may occur with severe disease (e.g., bed rest) or with BMI loss and muscle wasting that has also been shown to occur with SARS-CoV-2 infection. Muscle wasting can also cause systemic issues that may influence the bone. Medications used to treat SARS-CoV-2 infection also have a negative effect on the bone. Lastly, SARS-CoV-2 infection may also worsen conditions such as diabetes and negatively affect kidney function, all of which could contribute to bone loss and increased fracture risk.

**Summary:**

SARS-CoV-2 can negatively affect the bone through multiple direct and indirect mechanisms. Future work will be needed to determine what patient populations are at risk of COVID-19-related increases in fracture risk, the mechanisms behind bone loss, and therapeutic options. This review article is part of a series of multiple manuscripts designed to determine the utility of using artificial intelligence for writing scientific reviews.

## Introduction

**Fig. 1:**
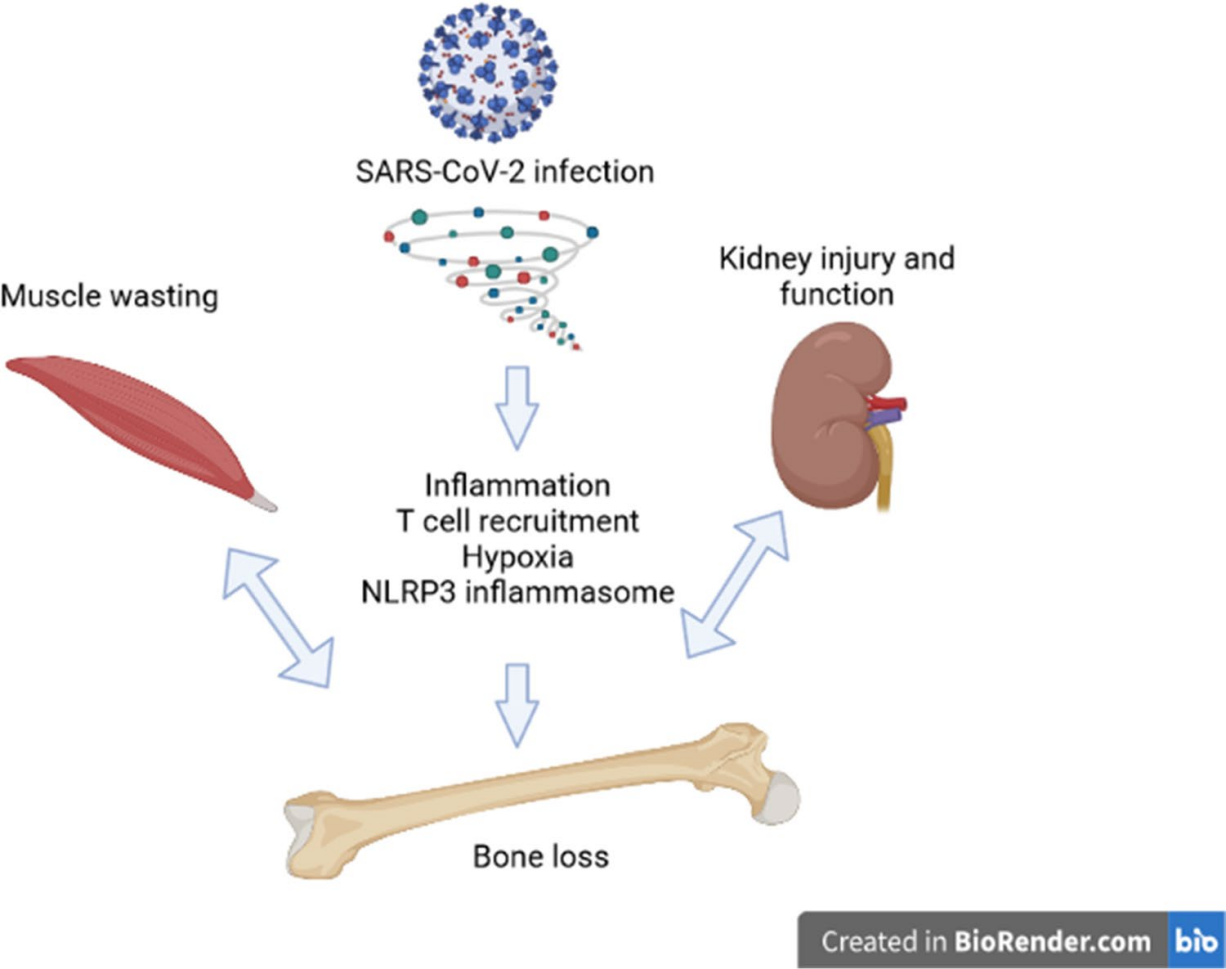
Overview of mechanisms through which bone loss can occur with SARS-CoV-2 infection. Created in BioRender

This is one of many articles evaluating the utility of using AI to write scientific review articles on musculoskeletal topics [1]. The first draft of this review was written entirely by humans. Refer to this edition’s Comment paper for more information [2]. SARS-CoV-2 is the novel coronavirus that resulted in the COVID-19 worldwide pandemic. According to the World Health Organization (WHO) COVID-19 Dashboard, as of November 2023, there have been 772,052,752 confirmed cases and COVID-19 has resulted in 6,985,278 deaths [3]. The COVID-19 syndrome includes a wide array of symptoms, but the most common include fever, cough, fatigue, shortness of breath, and other flu-like symptoms [4–7]. Much of the focus of the research has been on the lung damage that occurs with SARS-CoV-2 infection [8, 9]. However, in addition to the pulmonary effects, there are systemic effects such as joint pain (arthralgia), muscle pain (myalgia), and an increased risk of acute kidney injury (AKI) associated with the viral infection [10–12]. Disease severity in SARS-CoV-2 infection is dependent on several factors, including obesity, age, and pre-existing conditions such as diabetes [6, 13, 14]. A subset of patients infected with SARS-CoV-2 developed a syndrome called a “cytokine storm,” which is an acute increase in multiple inflammatory cytokines [15, 16]. This “cytokine storm” has been associated with the more severe forms of multiorgan dysfunction, and the specific elevated cytokine may vary depending on the examined tissue [17–19]. An estimated 10–30% of SARS-CoV-2 infection survivors will develop post-acute sequelae of COVID-19 (PASC) which is defined as having symptoms lasting beyond 8 weeks of initial infection [20]. PASC is a syndrome with a variety of multisystem manifestations, including but not limited to kidney failure, markers of tissue inflammation, local immune cell infiltration, and endothelial injury, while the musculoskeletal system can manifest with myalgia, joint pain, sarcopenia, and heterotopic ossification [20]. The full long-term and systemic effects of SARS-CoV-2 infection are under investigation as researchers and healthcare professionals determine comorbidities and treatment options for COVID-19 survivors. Among the systemic effects of SARS-CoV-2 infection already discussed, there is evidence that bones are affected by SARS-CoV-2 infection and patients may be at an increased risk of fracture [21, 22] through pathways outlined in Fig. 1*.* In this review, we will summarize the current literature on COVID-19 and bone, describe the possible risk factors, and suggest possible mechanisms through which bone loss may occur.

## Clinical Evidence of SARS-CoV-2 Infection-Related Bone Loss

### SARS-CoV-2-Infected Patients Have Alterations in Mineral Metabolism and Bone Turnover Markers

Observations from the pandemic indicate that bone loss and mineral metabolism may be altered with SARS-CoV-2 infection. Studies have indicated that hypocalcemia as measured from serum calcium levels is common amongst COVID-19 patients, similar to other viral infections including MERS-CoV and Ebola virus. An early study comparing 20 SARS-CoV-2-infected patients and 20 SARS-CoV-2-uninfected hospitalized patients indicated that hypocalcemia was more frequent among SARS-CoV-2-infected patients [23]. A different retrospective cohort study with 531 patients found 82% of patients exhibited hypocalcemia [24]. Patients were excluded based on conditions that could alter serum calcium levels, including chronic kidney disease (CKD), osteoporosis, and glucocorticoid use. This study connected hypocalcemia with more severe SARS-CoV-2 infection, with multivariate and univariate analysis indicating calcium levels were an independent risk factor associated with hospitalization [24]. Additionally, bone turnover markers may be altered, with one study showing serum levels of C-terminal telopeptide of type 1 collagen (CTX) (a bone degradation peptide) and osteocalcin (a marker of bone formation) were lower in SARS-CoV-2-infected patients compared to age- and sex-matched SARS-CoV-2-uninfected controls (*n* = 25/group), indicating reduced turnover [25••]. In this study, serum measurements were taken from hospitalized patients who were not bedridden or in the intensive care unit (ICU), indicating that even moderate SARS-CoV-2 infection alters bone turnover [25••]. The average age of patients was above 65 for both groups. However, further analysis will need to be done with more patients and including those who were not hospitalized. Overall, there is evidence that SARS-CoV-2 infection results in alterations to bone mineral metabolism and thus may alter fracture risk.

### Increases in Vertebral Fractures Occur with SARS-CoV-2 Infection

Vertebral fractures (VF), an indicator of poor bone quality and future risk of additional fracture, are highly prevalent in COVID-19 patients and those with VFs appear to have worse outcomes, indicating that bone loss may be concurrent with worse outcomes [26••]. This study utilized chest X-rays taken during hospital admission to detect VFs. Severity of the disease was determined by factors such as mortality and whether mechanical ventilation was required. Limitations of this study are the retrospective nature and the limited sample size. A subsequent study was conducted with more patients and confirmed that VFs were prevalent among SARS-CoV-2-infected patients [27]. However, this larger study also indicated that VFs were prevalent among SARS-CoV-2-uninfected patients admitted to the emergency department and that mortality risk was only significantly higher in COVID-19 patients with multiple fractures. Interestingly, additional work has indicated that hospitalized patients with VFs had lower respiratory function at their 6-month follow-up visit [28]. These initial studies show that patients with SARS-CoV-2 infection may be at risk of future fracture, but further work will be needed.

### Bone Mineral Density Is Correlated with Severity of SARS-CoV-2 Infection and May Be Lower in COVID-19 Patients

While bone mineral density (BMD) based on dual-energy X-ray absorptiometry (DXA) scans was not a practical measurement to take from those infected with SARS-CoV-2 during their illness, researchers have been able to calculate BMD from the chest CTs of those admitted to the hospital. Vertebral BMD calculated from the initial chest CTs of hospitalized SARS-CoV-2-infected patients (*n* = 58) showed a lower BMD was predictive of patients with higher rates of mortality, ICU admission, and mechanical ventilation using univariate analysis, indicating patients with low BMD would be more likely to have severe disease [29]. Of interest, this same study showed age was an equivalent predictor of outcomes when compared to BMD. In a separate study (*n* = 209), vertebral BMD and whether the patient was classified as having lower BMD (<100 Hounsfield Units) were significant independent predictors of mortality in SARS-CoV-2-infected patients using univariate analysis [30]. BMD correlated with the clinical classification of the severity of the SARS-CoV-2 infection and multivariate analysis also indicated that vertebral BMD and whether the patient was classified as having lower BMD were independent predictors of mortality [30]. Both studies utilizing vertebral BMD did not have initial BMD baselines prior to infection, so it remains to be seen if patients with low BMD are at higher risk of severe SARS-CoV-2 infection or if the initial immune response to SARS-CoV-2 infection alters BMD. However, one study found that chest BMD was decreased 81 days after hospital discharge compared to the BMD calculated at diagnosis among hospitalized SARS-CoV-2-infected patients (*n* = 58) [31]. Overall, these studies indicate that severe SARS-CoV-2 infection requiring hospitalization could cause BMD loss. However, there is evidence that even non-severe SARS-CoV-2 infections can lead to BMD loss. A study measured BMD with DXA from osteoporotic patients (*n* = 100) and compared these baseline scans in patients who had a SARS-CoV-2 infection and those who did not 9 months later and determined that the BMD was lower after 9 months in osteoporotic SARS-CoV-2-infected patients, but not osteoporotic SARS-CoV-2-uninfected patients. In this study, BMD was restored to baseline in patients with SARS-CoV-2 infection by 21 months; however, they did not show gains in BMD due to their osteoporosis treatment like the SARS-CoV-2-uninfected group. The SARS-CoV-2-infected group included those with mild, moderate, and severe infections. While those with severe infections had greater losses in BMD, BMD loss still occurred in those with mild and moderate infections [32]. While these previous studies determined BMD loss may occur, they did not investigate mechanisms for bone loss. One small study (*n* = 130) provides some possible mechanistic insight. Specifically, when comparing age- and body mass index (BMI)-matched SARS-CoV-2-infected patients 3-months post-infection with never-infected control patients, they found a significantly higher level of serum osteoprotegerin (OPG), a bone remodeling regulator, in the infected patients [33]. These SARS-CoV-2 patients also exhibited significant reductions in BMD compared to never-infected controls. While they found a significant inverse correlation between OPG levels and DXA T-score measurements, causation was not specifically examined. Of note, when comparing SARS-CoV-2-infected patients with never-infected controls, no differences were observed in serum angiotensin converting enzyme-2 (ACE-2) levels and no correlations were observed between ACE-2 levels and either OPG levels or DXA T-score measurements [33]. Another study examined the bone from total hip arthroplasties from controls, osteoporotic, and patients infected with SARS-CoV-2 at most 12 months prior to the total hip arthroplasties to determine if differences in bone quality, as measured by mechanics and lacunar geometry, existed [34•]. Femoral heads were tested with synchrotron image-guided failure assessment to determine structure and mechanics. There were no differences in modulus between SARS-CoV-2-infected patients and controls, while osteoporotic samples did show differences. There was a high amount of variability in the modulus of the SARS-CoV-2-infected patients, which could be an indication of early signs of mechanical deterioration or that some patients are susceptible to mechanical deterioration while others are not. However, artificial intelligence (AI)-guided image analysis indicated that SARS-CoV-2 infection had similar changes to lacunar structure as osteoporotic patients. Lacunae were larger and more spherically shaped. It is important to note that this study utilized a small number of biological samples. Control sample size was five females, osteoporotic sample size was five females, and SARS-CoV-2-infected sample size was five males and five females. Multiple test samples were taken from these patients, but biological sample size remained low [34•]. Further work will need to be done to determine the long-term effects of SARS-CoV-2 infection on BMD and fracture risk, especially in patients who were not hospitalized with SARS-CoV-2 infection. Furthermore, more information on the mechanism of how bone loss occurs will need to be determined through basic and clinical research.

## In Vitro and Preclinical Work Indicating that SARS-CoV-2 Infection Negatively Alters the Bone

### SARS-CoV-2 May Directly Infect Bone Marrow Cells

In vitro research is an invaluable tool to determine how SARS-CoV-2 affects the bone. SARS-CoV-2 infects cells primarily through the ACE2 receptor. Previous work during the SARS pandemic indicated that human monocytes, precursors to osteoclasts, express ACE2 through fluorescence-activated cell sorting (FACS) analysis [35]. This study also found that exposure of RAW264.7 cells, a murine macrophage line, to the SARS coronavirus protein 3a/X1 increased osteoclastogenesis. Additionally, osteoblasts and osteoclasts isolated from rodent calvaria and femur bones were shown to express ACE2, indicating the possibility of direct infection [36]. Exposure of in vitro cell lines to proteins associated with SARS-CoV-2 indicate that infection may result in changes to cellular differentiation within the bone marrow. In one study, researchers cultured murine bone marrow cells with SARS-CoV-2 spike protein and determined that macrophages could be infected [37]. Furthermore, changes occurred to the senescence-associated secretory phenotype (SASP). Spike protein upregulated the SASP response in young and aged male macrophages that were activated by macrophage colony stimulating factor (M-CSF). Additionally, tumor necrosis factor (TNF) α, interleukin (IL)-1β, and IL-6 RNA expression were upregulated with spike protein exposure in M-CSF induced macrophages isolated from young and old male mice. Cathepsin K expression was also higher in M-CSF-induced macrophages obtained from young and old male mice with spike protein exposure [37]. Cathepsin L expression was only higher in M-CSF-induced macrophages from aged males, whereas cathepsin B expression was higher in M-CSF-stimulated macrophages obtained from young male mice and lower in those obtained from aged male mice. There were fewer changes in IL-34-induced macrophages and in macrophages obtained from female mice and induced by either M-CSF or IL-34 [37]. In vitro evidence shows that SARS-CoV-2 can directly infect bone marrow cells, which can lead to alterations in cellular differentiation. Moreover, changes in cellular signaling can further alter the cellular composition of the bone marrow. Different variants of SARS-CoV-2 have arisen which affect transmission and disease severity [38]. To our knowledge, thus far, there has not been work directly comparing the effects of different variants of SARS-CoV-2 on bone cells in vitro.

### Preclinical Models of COVID-19 Show Alterations to Bone Structure and Osteoclast Numbers

To date, there have been few studies with preclinical models investigating the effects of SARS-CoV-2 infection on bone loss. Currently, SARS-CoV-2 studies must be conducted in Biosafety Level 3 (BSL3) facilities, which is limiting based on costs and expertise required to work in such facilities. Of further note, mice, the most used experimental animal, are not naturally susceptible to SARS-CoV-2 with intranasal infection [39]. One strategy to overcome this is to genetically modify mice to express human ACE2 (hACE2). However, this can result in the overexpression of hACE2 compared to humans or in tissues where it does not normally exist, and severity of the disease depends on the promoter for the gene [40, 41]. Adenoviruses can be utilized to infect regular mouse strains; however, this model may not demonstrate the extrapulmonary effects of the disease [42]. Animals can also be infected with murine forms of coronavirus [39]. Alternatively, animals such as golden Syrian hamsters have ACE2 receptors more like humans and can develop pneumonia-like symptoms with infection with human SARS-CoV-2 [43, 44].

Despite the hurdles involved, there is some information on bone in preclinical models available. Our group was the first to find that trabecular bone loss occurred with SARS-CoV-2 infection. In our study, K18-hACE2 mice were infected with 0, 1E3, 1E4, and 1E5 plaque-forming units (PFU) [45••]. Femoral trabecular bone loss was observed irrespective of disease severity and was due to decreases in trabecular number, as trabecular thickness remained the same. Osteoclastogenesis was increased as determined by histological analysis. Another group also found losses in vertebral trabecular bone volume fraction and increases in osteoclastogenesis with SARS-CoV-2 infection using this mouse model [46•]. Similar to this model, a different group has demonstrated trabecular bone loss within tibiae, femurs, and vertebrae of golden hamsters infected with SARS-CoV-2 [47••]. Here, hamsters showed trabecular bone loss as soon as 4 days post-infection (dpi) and up to 60 dpi. Osteoclast numbers and activity were increased as measured by histology. Additionally, this group observed increases in nuclear factor kappa beta ligand (RANKL), matrix metalloproteinase 9 (MMP9), and inflammatory cytokines IL-1β and TNF-α. While osteoprotegerin (OPG) was decreased [47••]. In contrast to findings from other groups, one group demonstrated decreased osteoclastogenesis, as measured by histology, with SARS-CoV-2 infection in the adenovirus (Ad) 5-hACE2 mouse model [48]. This group demonstrated that bone marrow macrophages were infected in this model through the neuropilin-1 (NRP1) receptor [48]. Bone structure was not reported and thus cannot be compared to previous results. The contrasting results are likely due to the differences in the models or the length of time since infection. Additionally, different virus strains were utilized as the hACE2 model studies utilized the US-WA1/2020 strain and the work with the Ad5-hACE2 model utilized the beta strain B.1.351. Further work will be needed to determine whether osteoclastogenesis is increased in humans with SARS-CoV-2 infection, if these effects are variant-dependent, whether bone loss persists or can recover over time as well as the length of time needed for the recovery, and which model best represents the human condition. Related to the variant-dependent effects, different variants of SARS-CoV-2 have varying severity [38] and thus their effects on the bone may differ. However, to our knowledge, there have been no preclinical studies directly comparing the effects of different variants of SARS-CoV-2 on the skeleton, which is an important consideration for future investigations.

## Cellular Mechanisms for Changes in Bone with SARS-CoV-2 Infection

### Inflammation

There are multiple possible mechanisms for how SARS-CoV-2 infection can alter the bone. Inflammation is known to be increased with COVID-19 patients [17–19]. Inflammation is necessary for the immune response to infection; however, chronic conditions of inflammation can result in negative effects on tissues. Inflammation can result in an increase in reactive oxidative species (ROS), which may cause protein damage, and an increase in advanced glycation end-products (AGEs) [49]. AGEs can affect protein function, resulting in crosslinks in bone that may have a brittling effect on bone [50]. Furthermore, activation of receptor for AGEs (RAGE) along with the inflammatory environment can result in increases in osteoclastogenesis [51], which may occur with COVID-19. Changes to the bone marrow microenvironment can lead to changes in bone composition and strength. Thus, inflammation may affect bone through its effects on bone cells and on the tissue directly.

### NLRP3 Inflammasome

The nucleotide binding oligomerization domain-like receptor containing pyrin domain 3 (NLRP3 inflammasome) has been studied as contributing to bone and joint disease [52–57]. The NLRP3 inflammasome regulates IL-1 through the regulation of caspase-1. Caspase-1 cleaves IL-1β into its active form, and caspase-1 signaling is tightly controlled by the NLRP3 inflammasome. The NLRP3 inflammasome is activated through priming signals such as TNF and then triggers signals such as ROS, Ca2+ influx, and K+ efflux. It also has been implicated in worsening conditions such as rheumatoid arthritis and osteoarthritis. Preclinical models have connected the NLRP3 inflammasome activation with bone loss. Specifically, NLRP3 inflammasome deficiencies have protected mice from ovariectomized (OVX)-related bone loss [58] and mice genetically altered to overexpress NLRP3 signaling have skeletal structural defects with thinner cortical bone and reduced trabecular bone volume [59]. In humans, IL-18, one of the downstream signaling events of the NLRP3 inflammasome, is elevated in patients with osteoporosis [60], and therapeutics targeting the NLRP3 inflammasome are of interest for osteoporosis treatment [61].

Some studies have shown that SARS-CoV-2 infection can activate the NLRP3 inflammasome. Initial work indicated that the interaction of ACE2 receptor with SARS-CoV-2 spike protein activates the NLRP3 inflammasome in human very small embryonic like stem cells (VSELs) and hematopoietic stem cells (HSCs) from human bone marrow, with NLRP3 expression as measured by quantitative PCR being higher with SARS-CoV-2 exposure, along with expression of IL1-β and IL-18 [62]. Another study has indicated that human monocytes produced caspase-1 in response to SARS-CoV-2 infection, and IL-1β production was elevated [63•]. Clinically, COVID-19 patients had higher concentrations of Casp1p20 and IL-18 in their serum and NLRP3 activation in their peripheral blood mononuclear cells [63•]. Immunohistochemistry indicated higher levels of NLRP3 in post-mortem tissues from COVID-19 patients compared to controls, and there were correlations with Casp1p20 and IL-18 and disease severity [63•]. Thus, this may be one mechanism through which SARs-CoV-2 affects bone.

### Th17 Cells

T helper 17 cells (Th17) are a subset of Th cells that produce IL-17 [64]. These differentiate from naïve T cells in the presence of transforming growth factor-beta (TGF-β) and inflammatory stimuli. Th17 cells recruit macrophages and other immune cells and are implicated in immune responses and autoimmune disorders [65]. Importantly, Th17 can regulate osteoclastogenesis through the release of IL-17 inducing RANKL [66] and may be involved in the fracture healing process through its effects on bone cells [67]. There is evidence that Th17 cells are involved in the body’s response to SARS-CoV-2 infection through regulating Th1, Th2, and regulatory T cells (Treg) cells [68]. There is a decrease in the concentrations of Th cells and Treg cells with COVID-19, likely indicating a dysregulated inflammatory response [69]. Additionally, IL-17 is overexpressed in patients with SARS-CoV-2 infection, indicating an increase in Th17 activity [70]. The upregulated Th17 activity could lead to the “cytokine storm” that is present in severe forms of COVID-19.

### Hypoxia

Hypoxia is a state of low oxygen and may influence bone health. Evidence shows that conditions such as anemia and chronic obstructive pulmonary disease are linked with low BMD [71–74]. Hypoxia can result in hypoxia-induced factors (HIF), acidosis, alterations in energy metabolism, ROS, and erythropoietin (EPO) production [71]. In vitro hypoxia conditions impair osteoblast proliferation, and animals exposed to hypoxic conditions show bone loss [75, 76]. In contrast, osteoclast formation and activity is stimulated by hypoxic environments. It should be noted that this occurs with intermittent hypoxic conditions as continual hypoxia results in cell death. Acidosis limits the ability of osteoblasts to mineralize their environment, while ROS impairs osteoblastogenesis and increases osteoclastogenesis. There has been conflicting evidence that EPO can stimulate bone formation [77, 78]; however, this may only be at above physiological levels or in the absence of enhanced osteoclastogenesis as others have observed bone loss with EPO [71, 79, 80]. Previous evidence indicates OVX rats exposed to a hypoxic environment showed bone loss that exceeded the loss due to OVX alone [81]. Notably, SARS-CoV-2 infection can cause hypoxia in patients due to low oxygen levels that can occur with the disease. This will be of particular interest in patients where disease severity required mechanical ventilation. Additionally, some patients may have silent hypoxia, where the patients have lower oxygen saturation levels but do not experience difficulty breathing [82]. Furthermore, obesity, a common comorbidity of severe SARS-CoV-2 infection, may increase the hypoxia in COVID-19 patients [83]. Therefore, hypoxia may be one method by which SARS-CoV-2 infection reduces bone, by increasing osteoclastogenesis and/or impairing osteoblastogenesis/osteoblast proliferation, especially in patients with severe SARS-CoV-2 infection.

### RANK/RANKL/OPG

Receptor activator of nuclear factor kappa B (RANK)/RANKL/OPG is an important regulatory signaling pathway in osteoclastogenesis, with RANKL inducing osteoclast differentiation. OPG serves as a decoy receptor for RANKL and suppresses osteoclastogenesis [84]. It is released by osteocytes as well as osteoblasts and is a mechanism by which osteocytes coordinate bone remodeling. RANKL is also expressed on immune cells and is important for the development of immune organs [84]. As such, this may be altered with SARS-CoV-2 infection. RANK/RANKL signaling is also important with muscle development. One study investigating periodontitis indicated increases in RANKL in the saliva of COVID-19 patients as measured by ELISA [85]. Another study looking at human serum of COVID-19 patients showed that the RANKL/OPG ratio was increased due to decreases in OPG [86]. In contrast, a different study has indicated that BMD is lower in patients with SARS-CoV-2 infection and that OPG was higher with SARS-CoV-2 infection [33]. RANKL was not measured, so the ratio cannot be determined. Higher OPG levels may be a reaction to an increase in osteoclast activity in order to maintain bone homeostasis. While contradictory results require further exploration, changes in RANK/RANKL/OPG may be an indicator of which COVID-19 patients will experience bone loss.

## Indirect Effects of SARS-COV-2 Infection on Bone

### Muscle Weakness and Mechanical Unloading

Muscle loading affects the bone as the bone responds to the forces placed upon it and muscle-bone crosstalk occurs [87]. Myalgia, or muscle aches, are a common complication of SARS-CoV-2 infection. Additionally, myositis or muscle inflammation as defined by elevation in serum creatinine kinase (CK) can occur in patients is associated with poor outcomes such as ICU-admission [5, 88–91]*.* Results with nuclear imaging of patients with severe CK elevation shows that the myositis associated with the infection is diffuse and not localized to one area of the body [92]. Thus far, there has not been evidence suggesting direct infection of muscle tissue with SARS-CoV-2 and no single cytokine pathway has been associated with the inflammation [93]. Only one autopsy study of patients that died during the acute infection detected SARS-CoV-2 RNA in some muscle tissue, but did not observe virus present by immunohistochemistry or electron microscopy [94]*.* The diffuse myositis observed with SARS-CoV-2 infection and the failure to consistently identify viral particles in myocytes suggests that the myositis is a result of the “cytokine storm” brought on by the infection. When the myositis is severe, the myoglobin released from muscle breakdown can cause a form of kidney failure referred to as rhabdomyolysis [95–98]. The effects of acute kidney failure with SARS-CoV-2 infection will be discussed in a later section.

Of note, muscle wasting and decreased muscle strength were observed in ICU-admitted SARS-CoV-2-infected patients as early as 1 day after admission [99••]. Unfortunately, the muscle atrophy associated with SARS-CoV-2 infection cannot be improved with aggressive physical therapy [100]. In one study that examined 23 SARS-CoV-2-infected ICU patients, they found that 69% of these patients had limb weakness 1 month into their recovery [100]. Ultrasound measurements of the dominant leg medial gastrocnemius in 259 patients recovering from SARS-CoV-2 observed lower muscle thickness and high muscle stiffness [101]. This study also recorded reduced muscle strength as measured separately by hand grip strength one month after hospital discharge [101]. Additionally, another study found that patients recovering from SARS-CoV-2 had reduced physical function up to 6 months after hospital discharge and handgrip strength at 1 month predicted those with complications at 6 months [102]. Others have reported that muscle aches are a common symptom after COVID-19 [103, 104] and that myalgia is a predictor of those who will have PASC [105]. Through muscle wasting, bone may also be indirectly affected through SARS-CoV-2 infection effects on muscle. Of note, muscle weakness and unusual muscle pain are common complaints of patients developing PASC [104–106].

### Systemic Effects of Muscle Breakdown

The multiorgan dysfunction associated with SARS-CoV-2 infection may adversely affect skeletal muscle mitochondria. Studies in patients with non-SARS-CoV-2 sepsis and multiorgan dysfunction observed mitochondrial alterations via multiple metabolic pathways [107–109]. These same studies found that survival from the septic event was associated with the improvement in mitochondrial function [107–109]. Recovery from sepsis does not result in full recovery of mitochondrial function. Residual muscle weakness is associated with the failure of mitochondrial functional recovery [110]. Through these multiple mechanisms, muscular weakness both acutely and chronically may contribute to bone loss after recovery from SARS-CoV-2 infection.

### Nutritional Deficiencies

Concerns for overall nutrition status and isolated nutritional deficiencies are common in all forms of sepsis. Loss of appetite has been observed in COVID-19 patients [111]. Weight loss associated with SARS-CoV-2 infection is frequently encountered [112, 113]. Loss of at least 5% of pre-infection weight was observed in 29% of patients with severe and mild SARS-CoV-2 infection [113]. The mean median BMI loss was 2.3% in the affected patients and was independent of hospitalization but correlated with the length of symptoms [113]. BMI is correlated with the mechanical load placed upon the skeleton and will thus affect bone mass. The weight loss is not limited to the acute infection. A long-term follow-up study investigated the nutritional status of 407 patients after they were hospitalized for SARS-CoV-2 and found losses in appetite, early satiety, and decreased taste for up to 5 months after discharge in 30% of the patients [112]. Such conditions can lead to continued weight loss and decreases in bone mass during recovery.

A study early in the COVID-19 pandemic identified deficiencies in vitamin D and trace metals in patients admitted to the hospital [114]. This study of 150 COVID-19 patients reported that 76% of those patients exhibited low vitamin D levels. Subsequent studies of vitamin D status and SARS-CoV-2 infections found that higher levels were associated with faster clearance of the virus based on nucleic acid negative conversion time measured from throat swab samples [115]. A population study also observed that low vitamin D levels were correlated with the occurrence of SARS-CoV-2 infections [116]. Vitamin D deficiencies are associated with dysregulation of the immune system and increases in inflammatory markers [117]. Of note, levels of Treg can modulate inflammatory responses to sepsis, and low levels of Treg are associated with the “cytokine storm” that can occur with SARS-CoV-2 infection [118]. Vitamin D supplementation has been found to increase Treg levels [119]. Unfortunately, this finding has not been observed in a systemic review of research evaluating vitamin D supplementation and improvement in Treg levels [120]. On the other hand, vitamin D levels are inversely correlated with inflammatory cytokine TNF-alpha levels in healthy women [121]. Lower vitamin D levels were also found to be associated with higher TNF-α and IL-6 levels in patients with inflammatory bowel syndrome [122]. Replacement of vitamin D in septic patients in the ICU has not been previously shown to be beneficial [123]. Along these lines, low vitamin D levels during initial infection was also associated with the development of PASC [124]. Additionally, vitamin D remains lower in patients experiencing PASC [125••]. However, supplementation of vitamin D was not found to protect against SARS-CoV-2 infections in healthcare workers [126].

With regard to trace elements, zinc and selenium, they have been found to be low in patients with SARS-CoV-2 infections based on the meta-analysis of multiple articles [127]. Zinc and selenium are both important in bone metabolism with zinc deficiency being associated with imbalances in the RANKL/RANK/OPG pathways, resulting in bone reabsorption [128] and selenium affecting bone via multiple pathways [129]. To date, replacement of these trace elements have not been shown to improve outcomes in SARS-CoV-2-infected patients [130].

### Steroid Utilization

Corticosteroid usage has been shown to decrease disease severity and mortality in SARS-CoV-2 infection [131]. Glucocorticoids have direct effects on bones that reduce BMD through increases in osteoclasts, decreases in osteoblasts, and apoptosis of osteocytes that maintain the matrix [132]. There is also evidence that glucocorticoids cause disorganization of osteocyte lacunocanalicular networks and decrease osteocyte perilacunar remodeling [133, 134]. Glucocorticoids stimulate the M-CSF and RANKL pathways that result in increases in osteoclast formation [135]. As noted earlier, the spike protein of the SARS-CoV-2 virus has been shown in macrophages stimulated by M-CSF to accelerate the “cytokine storm” of the infection [37, 136]. The use of steroids in SARS-CoV-2 infection may therefore have an additive deleterious effect on BMD. Further studies will be needed to separate the effects of glucocorticoid treatment and the direct effects of SARS-CoV-2 virus infection.

## Concurrent Conditions and Severity of COVID-19

### Obesity and Diabetes Mellitus

In the outbreak of COVID-19 in New York, the second and third most common co-morbidities associated with severe disease and death were obesity and diabetes mellitus (DM) [6]. Early studies from China identified diabetes as a risk factor for severe COVID-19 syndrome, but because of the difficulty clinically defining obesity; the two diagnoses may be combined [6, 137]. Diabetes has been shown in multiple studies to increase the severity and mortality associated with SARS-CoV-2 infection [138, 139]. The association of obesity and insulin resistance is a well-recognized phenomenon [140]. Meta-analysis of studies comparing fractures and both type 1 and type 2 diabetes found an increased risk of hip fractures compared to the general population [141]. A similar meta-analysis of studies assessing the risk of hip fracture and increased BMI observed a significant increase in fractures in patients with a BMI of 25 kg/m^2^ compared to patients with BMI of 20 kg/m^2^ [142]. Obesity and type 2 DM are associated with higher levels of TNF-α and IL-6 [49]. These inflammatory markers are central to the “cytokine storm” of SARS-CoV-2 infection and increased osteoclast activity. The risk of developing type 1 and type 2 DM increases after SARS-CoV-2 infection [143], which could lead to subsequent losses in bone quality.

### Acute Kidney Injury and Chronic Kidney Disease

SARS-CoV-2 infection has been demonstrated to cause an increased incidence of AKI compared to uninfected hospitalized patients [144, 145]. AKI can lead to chronic kidney disease (CKD) [146]. Patients that survive AKI associated with SARS-CoV-2 infections are at high risk for CKD, especially those with severe disease and prolonged AKI recovery periods [147••]. CKD has a well-described negative affect on bone quality through multiple pathways [148]. Additionally, AKI that requires temporary dialysis has been shown to increase the risk of fractures; however, the pathogenesis is not well understood [149]. Of note, the kidney is integral for calcium and phosphorus homeostasis and loss of kidney function can result in mineral disorders that affect bone health. With progressive CKD, absorbed dietary phosphorous cannot be removed by the kidneys and this increase in serum phosphorous causes a decrease in serum calcium [150]. The increased serum phosphorous increases osteocyte and osteoblast production of fibroblastic growth factor-23 (FGF-23). The FGF-23 in turn inhibits renal reabsorption of filtered phosphorous [151]. Additionally, FGF-23 suppresses the 25-hydoxylation of vitamin D [152]. CKD is also known to alter mineral homeostasis due to lower serum calcium and 1,25-vitamin D levels, which stimulate the parathyroid glands to secrete parathyroid hormone (PTH). Increased PTH stimulates osteoclastic bone resorption releasing calcium into the serum [153]. Further, activity of 1α-hydroxylase CYP27B1 in the proximal tubule of the kidney as a result of nephron loss in CKD reduces 1,25-vitamin D formation [154, 155]. Moreover, animal models of progressive kidney disease found increased levels of activin A, which induces fibrosis. At the same time, activin A stimulates RANKL and the increase in osteoclastic bone resorption [156, 157]. Although this is an animal model, the similarities of activin A and SARS-CoV-2 stimulation of RANKL warrant additional study in humans. Taken together, the effects of SARS-CoV-2 infection on the kidney may result in the alterations in bone metabolism due to the calcium, phosphorous, FGF-23/Klotho, and parathyroid gland interrelationships.

## Conclusions

There is early evidence indicating that bone mass is altered after SARS-CoV-2 infection via multiple physiologic mechanisms. Many identified pathways are interconnected, with inflammation serving as a common link in multiple signaling cascades such as the RANKL/OPG pathway, the NLRP3 inflammasome, and Th17 cells. Additionally, the inflammatory response may also worsen other disease states such as diabetes and impaired kidney function. Therefore, it is unlikely that a single factor will be implicated as the cause of low BMD or increased fracture risk as a result of SARS-CoV-2 infection. Further investigations will be needed to determine if this bone loss is prolonged after recovery and if bone health is a factor in PASC. Moreover, the effect of different variants of SARS-CoV-2 will need to be investigated. Clinical evidence and appropriate preclinical models need to be developed to better understand the association between SARS-CoV-2 infection and alterations to bone health.

## Data Availability

Data available upon reasonable request.
